# Proteomic Analysis Reveals the Dynamic Role of Silicon in Alleviation of Hyperhydricity in Carnation Grown In Vitro

**DOI:** 10.3390/ijms19010050

**Published:** 2017-12-24

**Authors:** Sowbiya Muneer, Hao Wei, Yoo Gyeong Park, Hai Kyoung Jeong, Byoung Ryong Jeong

**Affiliations:** 1 Division of Applied Life Science (BK21 Plus program), Gyeongsang National University, Jinju 52828, Korea; sobiyakhan126@gmail.com (S.M.); oahiew@gmail.com (H.W.); jhksmile@naver.com (H.K.J.); 2 Institute of Agriculture and Life Science, Gyeongsang National University, Jinju 52828, Korea; iuyiuy09@naver.com; 3 Research Institute of Life Science, Gyeongsang National University, Jinju 52828, Korea

**Keywords:** carnation, hyperhydricity, immunoblots, mass spectrometer, proteomics, stress response, silicon

## Abstract

The present study depicted the role of silicon in limiting the hyperhydricity in shoot cultures of carnation through proteomic analysis. Four-week-old healthy shoot cultures of carnation “Purple Beauty” were sub-cultured on Murashige and Skoog medium followed with four treatments, viz. control (–Si/–Hyperhydricity), hyperhydric with no silicon treatment (–Si/+Hyperhydricity), hyperhydric with silicon treatment (+Si/+Hyperhydricity), and only silicon treated with no hyperhydricity (+Si/–Hyperhydricity). Comparing to control morphological features of hyperhydric carnations showed significantly fragile, bushy and lustrous leaf nature, while Si supply restored these effects. Proteomic investigation revealed that approximately seventy protein spots were differentially expressed under Si and/or hyperhydric treatments and were either up- or downregulated in abundance depending on their functions. Most of the identified protein spots were related to stress responses, photosynthesis, and signal transduction. Proteomic results were further confirmed through immunoblots by selecting specific proteins such as superoxide dismutase (SOD), ascorbate peroxidase (APX), catalase (CAT), PsaA, and PsbA. Moreover, protein–protein interaction was also performed on differentially expressed protein spots using specific bioinformatic tools. In addition, stress markers were analyzed by histochemical localization of hydrogen peroxide (H_2_O_2_) and singlet oxygen (O_2_^1–^). In addition, the ultrastructure of chloroplasts in hyperhydric leaves significantly resulted in inefficiency of thylakoid lamella with the loss of grana but were recovered in silicon supplemented leaves. The proteomic study together with physiological analysis indicated that Si has a substantial role in upholding the hyperhydricity in in vitro grown carnation shoot cultures.

## 1. Introduction

Carnation (*Dianthus caryophyllus* L.) is a unique attractive cut flower throughout the world due to its aesthetic beauty such as different colors, shapes, fragrance and number of varieties [1,2]. To attain an outstanding quality of plants such as being free from viruses, diseases, or other pests, the “plant tissue culture” practice is commonly used [3,4]. Although in vitro tissue cultured plants produce exceptional plant qualities, but the environmental conditions are not natural, which lead to several physiological variations, among which the most common is hyperhydricity [5,6,7].

Hyperhydricity is a serious threat to carnation due to various malfunctions which indeed can be seen by naked eyes such as fragile, dense, frizzy and translucent leaves [8,9]. The other physiological changes revealed during hyperhydricity are under developed cuticles, reduced number of palisades and sponge parenchyma [10,11,12]. Some important biochemical reactions such as photosynthesis, transpiration and plant hormones also change during hyperhydricity [13,14,15,16]. Hyperhydricity generates reactive oxygen species (ROS) in apoplast, chloroplasts, and mitochondria in the form of a singlet oxygen (O_2_^1^^–^), hydrogen peroxide (H_2_O_2_), and hydroxyl radical (^·^OH) [13]. The generation of ROS by hyperhydricity has been reported in many plant species including Arabidopsis, apple, garlic as well as carnation [17,18,19,20]. However, it is well known that, during oxidative damage induced by ROS, antioxidant enzymes like superoxide dismutase (SOD), catalase (CAT), ascorbate peroxidase (APX), and glutathione reductase (GR) are activated [21,22,23] to suppress the negative effects on plants.

Silicon (Si) is considered as the second utmost abundant element in the soil after oxygen and, because of its copiousness, all plant roots encompass some quantity of Si [24]. Silicon is transported in plants in the form of silicic acid (Si(OH)_4_) (below pH 9), which is an uncharged molecule [25], whereas, plants significantly diverge in their capability for Si uptake and transport because, other than monocots, most dicots are unable to accrue Si in their shoots and are thus included in the Si-excluder category [25]. Nevertheless, the Si-excluder plants have also shown their capacity to hoard Si in their shoots [26]. Moreover, to many life forms such as diatoms, silicon is an essential element required for the production of structural material [27]. Although it is considered as a non-essential element in higher plants, this element is routinely supplied to several crops like rice and sugarcane to obtain high and sustainable crop yield [28]. In addition, it has also been observed that Si has a dynamic role in alleviating biotic and abiotic stress in plants such as pests and pathogens [29,30], metal toxicity [31,32,33,34,35,36,37], drought stress [38,39], mineral deficiency [40], salt stress [41,42], and UV-B stress [43]. The positive effects of Si have been largely observed in several crop plants such as wheat, barley, rice, cucumber, tomato, and soybean [44,45,46,47].

In response to hyperhydricity stress, several approaches are being used for many years to prevent hyperhydricity such as a commonly-used temporary immersion system (TIS) [48,49]. Recently, we also observed that red and blue light emitting diodes significantly mitigated the hyperhydricity in different carnation cultivars [50]. As where stress levels and tolerance are concerned, Si has been proven a beneficial element to alleviate abiotic stresses [29,30,31,32,33,34,35,36,37,38,39,40,41,42,43]. Thus, to improvise the previous studies on silicon efficiency, we hypothesize that hyperhydricity can be ameliorated by supplementation of silicon in in vitro grown carnation. To follow our objective, we assessed the proteomics of shoot cultures of carnation involving the 2-DE and mass spectrometer to analyze the protein profile exposed to hyperhydricity and Si supply. Subsequently, the stress responsive proteins such as superoxide dismutase (SOD), ascorbate peroxidase (APX), and catalase (CAT), including photosynthetic proteins like PsaA and PsbA obtained from proteomic results, were further confirmed by immunoblots. Moreover, the levels of hyperhydric stress were observed by two stress markers viz. hydrogen peroxide (H_2_O_2_) and singlet oxygen (O_2_^1–^) through histochemical localizations. Furthermore, ultrastructure of chloroplast was studied using transmission electron microscopy (TEM).

## 2. Results and Discussion

### 2.1. Morphological Changes and Si Concentration in Response to Hyperhydricity and Si Supply

**Figure 1:**
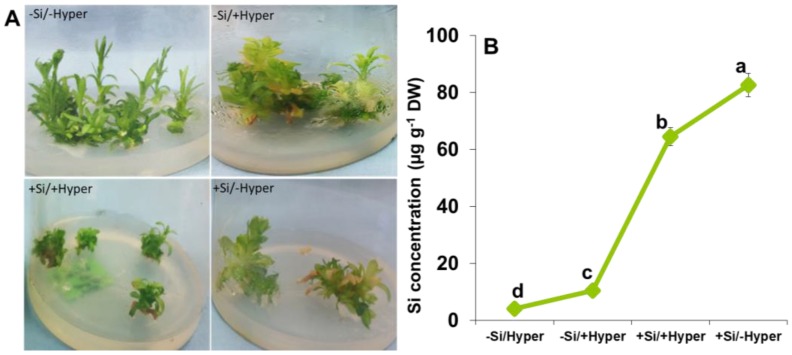
(**A**) Representative pictures (**B**) silicon up-take under hyperhydricity and silicon (Si) treatments (−Si/−Hyperhydricity; −Si/+Hyperhydricity; +Si/+Hyperhydricity; +Si/−Hyperhydricity) in shoot cultures of carnation (*Dianthus* *caryophyllus* L.) grown in vitro in Murashige and Skoog medium.

The hyperhydricity (−Si/+Hyperhydricity) leads to significant changes in micropropagated carnation shoot cultures compared to non-hyperhydric (−Si/−Hyperhydricity, control) (Figure 1A). The lengths of the shoots in −Si/+Hyperhydric were somewhat shorter with chlorotic, brittle, thick, curled and translucent leaves as also observed previously in hyperhydric micropropagated plants [8,9,51]. However, the hyperhydric shoot cultures supplied with silicon (+Si/+Hyperhydric) recovered the negative effects. The results obtained from morphological experiments depicted that exogenous Si supply plays an important role to mitigate hyperhydricity in shoot culture of carnation. Thereafter, Si was estimated in shoot cultures of carnation among all treatments to check its concentration for further analysis (Figure 1B).

### 2.2. Oxidative Stress in Response to Hyperhydricity and Si Supply

**Figure 2:**
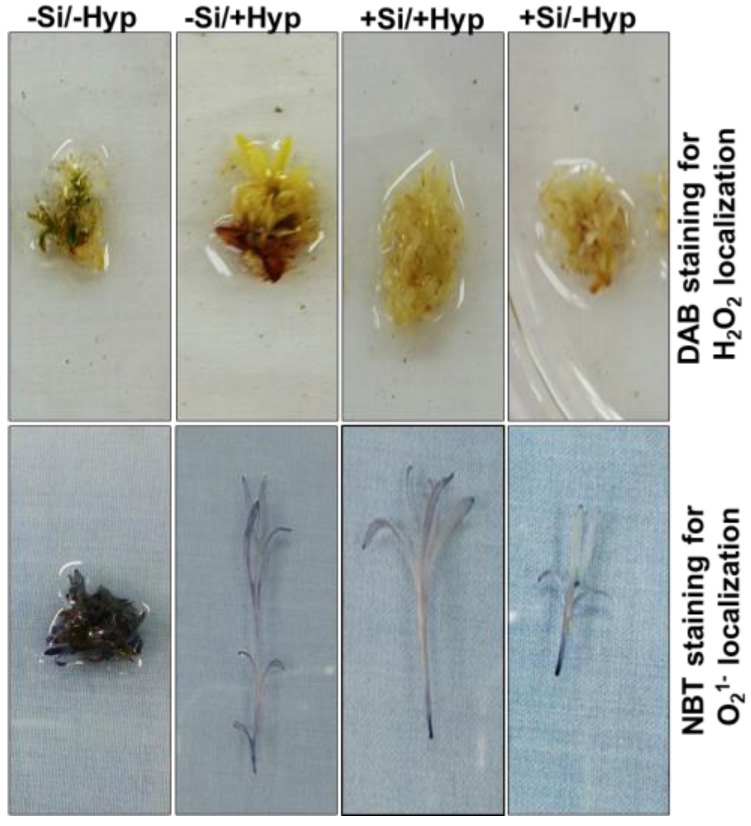
3,3′-diaminobenzidine (DAB) nitro-blue tetrazolium (NBT) mediated localizations of H_2_O_2_ and O_2_^1-^ under hyperhydricity and silicon (Si) treatments (−Si/−Hyperhydricity; −Si/+Hyperhydricity; +Si/+Hyperhydricity; +Si/−Hyperhydricity) in shoot cultures of carnation (*Dianthus* *caryophyllus* L.) grown in vitro.

Oxidative stress was observed in leaves of carnation shoot cultures by histochemical localizations of H_2_O_2_ and O_2_^1–^. The H_2_O_2_ and O_2_^1–^ localizations were observed as brownish and bluish layers in shoot cultures under hyperhydricity (−Si/+Hyperhydricity) (Figure 2). For hyperhydric shoot cultures supplemented with Si (+Si/+Hyperhydricity), the formation of brownish and bluish layers was reduced. A close relationship between hyperhydricity and oxidative stress is very common [9,11] as also shown in the present results. The results were in agreement with previous results reported in several in vitro grown hyperhydric plants [9,20,21]. In addition, the shoot cultures supplied with Si (+Si/−Hyperhydricity) were more stainless than overall treatments, which represents that Si is beneficial in other ways to shoot cultures of carnation alongside limiting the oxidative stress caused by hyperhydricity.

There are many reasons that Si improves the formation of ROS in carnation shoot cultures. Firstly, Si is transported to many plant parts and is deposited in cell epidermis or cortex of vascular tissues [41]. This led the cells to cell wall hardening, which could prevent the plants from external abiotic stress. Secondly, its polymerization causes extension of the cell wall and cell turgidity, which could help the hyperhydric plants to transpire properly and even could help them for normal stomatal opening and closure. In addition, Si can activate antioxidant enzymes to overcome abiotic stress including hyperhydricity as described in previous reports [41,42,43].

### 2.3. Carnation Proteome Changes in Response to Hyperhydricity and Si Supply

**Figure 3:**
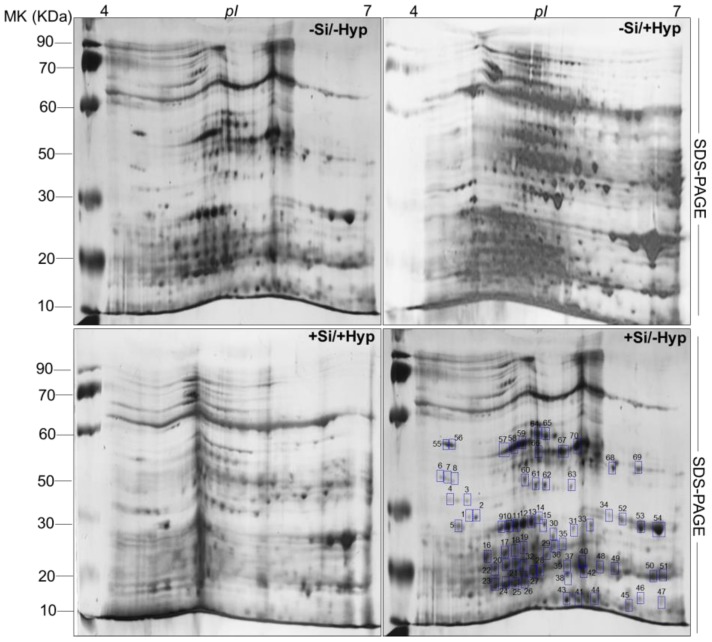
Representative second dimensional gels under hyperhydricity and silicon (Si) treatments (−Si/−Hyperhydricity; −Si/+Hyperhydricity; +Si/+Hyperhydricity; +Si/−Hyperhydricity) in shoot cultures of carnation (*Dianthus* *caryophyllus* L.) grown in vitro.

The comparative proteomic analysis was analyzed using IPG strips (*pI* 4–7, 13 cm) for first dimension and SDS-PAGE (sodium dodecyl sulfate polyacrylamide gel electrophoresis) for second dimension (Figure 3). The resulting 2DE maps from all four of the treatments were comparatively analyzed using progenesis software (Table S1) and the results of progenesis software revealed that protein folds under hyperhydricity (−Si/+Hyperhydricity) were either upregulated or downregulated compared to control (−Si/−Hyperhydricity). The protein folds in other two treatments, viz. +Si/+Hyperhydricity and +Si/−Hyperhydricity, showed a similar response as observed in control (−Si/−Hyperhydricity) (Table S1). The comparative analysis thus persuades the reader that Si supply is expedient for shoot cultures of carnation as it has ability to relieve hyperhydricity stress to a certain extent. Overall, the comparative analysis under four treatments revealed that 900 protein spots were reproducibly detected on 2DE maps, and, among 900 protein spots, 70 protein spots were differentially expressed (Figure 3), which were then identified by a MALDI-TOF MS (matrix assisted laser desorption/ionization time of flight).

**Figure 4:**
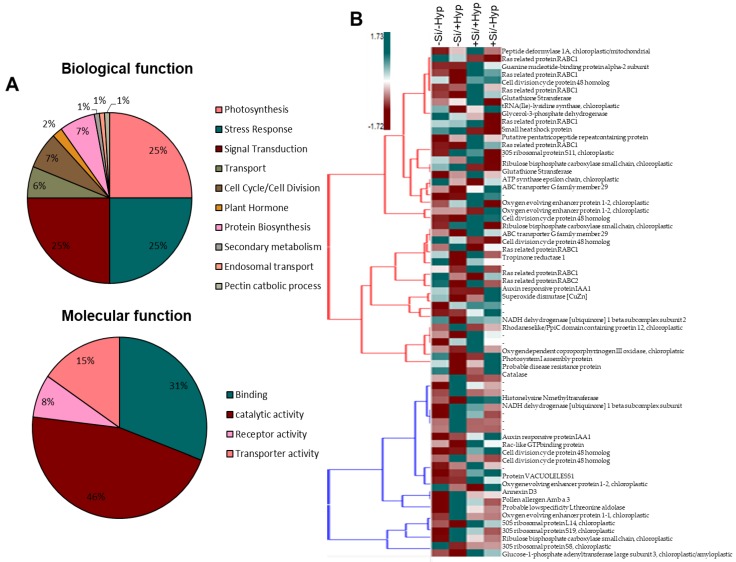
(**A**) functional classification of identified proteins and (**B**) heatmap of the differentially expressed protein spots under hyperhydricity and silicon (Si) treatments (−Si/−Hyperhydricity; −Si/+Hyperhydricity; +Si/+Hyperhydricity; +Si/−Hyperhydricity) in shoot cultures of carnation (*Dianthus* *caryophyllus* L.) grown in vitro.

The abundant proteins on 2DE maps were manually picked and digested with a trypsin enzyme. All of the digested protein in the form of peptides was evaluated on MALDI-TOF MS. The spectra obtained from MALDI-TOF were further searched online in matrix science (www.matrixscience.com) for protein identification and ion search (Table 1). The results indicate that most protein spots identified under four treatments of hyperhydricity and Si supply had a significant relationship to stress response, photosynthesis, and signal transduction using gene ontology (www.geneontology.com) and the rest of the categories were depicted as either primary or secondary metabolic processes (Figure 4A). Moreover, a heatmap was generated to distinguish general expression of differentially expressed protein spots (Figure 4B). The active changes in the abundance of 70 differentially protein spots clearly expressed the four treatments used in our experiments. The results indicated proteome changes under hyperhydricity (−Si/+Hyperhydricity) compared to control (−Si/−Hyperhydricity) and instincts that supply of Si (+Si/+Hyperhydricity) to hyperhydricity is beneficial to recover the stress.

#### 2.3.1. Proteins Related to Stress Response

**Figure 5:**
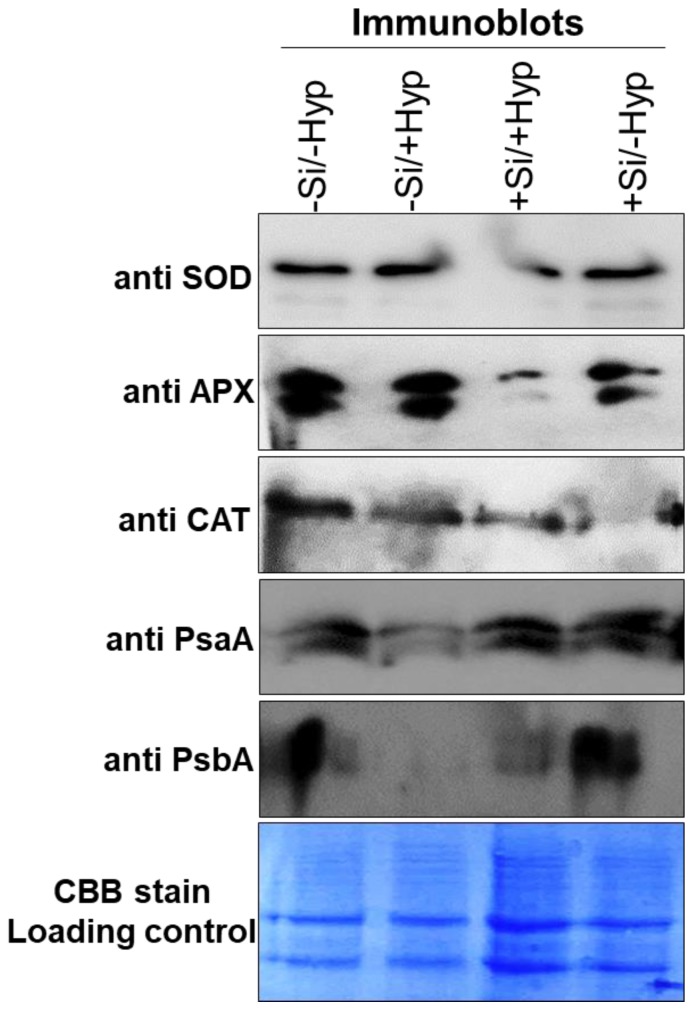
Immunoblots of superoxide dismutase (SOD), ascorbate peroxidase (APX), catalase (CAT), PsaA, and PsbA under hyperhydricity and silicon (Si) treatments (−Si/−Hyperhydricity; −Si/+Hyperhydricity; +Si/+Hyperhydricity; +Si/−Hyperhydricity) in shoot cultures of carnation (*Dianthus* *caryophyllus* L.) grown in vitro.

The stress responsive proteins play an important role in redox status or tolerance to abiotic and abiotic stresses [9,22,52]. It is well known that, during the ROS formation in plants, stress responsive proteins abundantly overexpress their expression to combat oxidative stress. In the present study, 25% of the protein spots were identified as a stress response protein. All of the identified stress proteins changed their abundance differentially among all treatments (Figure 3 and Figure 4, Table S1). The well-known stress responsive proteins that play an important role in ascorbate-glutathione cycle, viz. superoxide dismutase (SOD-Cu-Zn) and catalase (CAT), were identified in addition other stress responsive proteins (Table 1), previously also observed under hyperhydricity [9]. Moreover, a well-known stress response protein i.e., a heat shock protein (HSP) were also identified. The increased abundance of these stress responsive proteins to hyperhydricity (−Si/+Hyperhydricity) suggested a strong correlation between stress and tolerance because these proteins substantially reduce the oxidative stress. Meanwhile, in response to Si supply under hyperhydric conditions (+Si/+Hyperhydricity), the abundance of these stress responsive proteins was abundantly decreased. The decreased abundance of these stress responsive proteins indicated that Si has a potential role in alleviating the stress caused by hyperhydricity, which we also confirmed by immunoblots (Figure 5).

#### 2.3.2. Proteins Related to Photosynthesis

Photosynthesis is a key energy source for plants and the efficiency of photosynthesis can be significantly reduced by biotic or abiotic stress. The interference in photosynthesis to abiotic stress occurs mainly during non-cyclic photophosphorylation during hydrolysis of water in reaction center of PSII and during electron transfer in the reaction center of PSI. The effect of abiotic stress on photosynthetic proteins is well known such as drought, salinity and cold [53,54,55,56]. The proteins involved in this class correspond to 25% of all differentially represented proteins under hyperhydric and Si supplemented carnation shoot cultures. Several proteins involved in photosynthesis such as Ribulose bisphosphate carboxylase small chain, chloroplastic (RuBisCO), Oxygen evolving enhancer protein 1-2, chloroplastic, and Photosystem I assembly protein was decreased in abundance under hyperhydricity (−Si/+Hyperhydricity) (Table 1 and Table S1), whereas, under Si supply, the abundance of these proteins were increased, which was as also confirmed by immunoblots (Figure 5) of two photosynthetic proteins PsaA and PsbA. The decreased abundance of these photosynthetic proteins in shoot cultures of carnation to hyperhydric stress might be due to poor stomatal opening/closing or leaf abnormalities (brittle leaves, mineral deficiency) as also seen in our morphological data (Figure 1). The other reasons for the decrease in abundance of photosynthetic proteins might be a lack of proper photosynthesis due to poor development of chloroplast, whereas, an increase in abundance of these proteins due to exogenous Si obviously recovered the negative effects by reducing the brittle and shaggy appearance of carnation leaves and indeed increasing the photosynthetic activity. 

#### 2.3.3. Proteins Related to Signal Transduction

Signal transduction network plays a vital role in plant growth and development [57,58]. There are several signals in plants that control many important biochemical processes such as plant hormones, light perception, plant pathogen, stress levels, cell divisions, and circadian rhythms. All of these biochemical processes in plants respond in numerous ways when exposed to environmental conditions. Among the environmental conditions, abiotic stress is most common in plants and all signaling network changes according to plant’s ability to cope with these stress conditions. Our proteomic results revealed that 25% of proteins were identified that were related to signal transduction (Figure 4A). The proteins involved in signal transduction, such as ras related protein RABC1 and glutathione S-transferase, were significantly increased in shoot cultures of carnation under hyperhydricity (−Si/+Hyperhydricity) (for fold changes, please see Table S1). However, when Si was supplied to hyperhydric shoot cultures, the proteins belong to signal transductions were abundantly decreased. 

### 2.4. Validation of Selected Differentially Expressed Proteins

The activities of some proteins identified in this current work involved in stress response and photosynthesis were analyzed to validate the proteomic results. To validate the proteomic data, there are several reliable techniques; taking protein expressions into consideration, immunoblotting was used, which has been used in much proteomic data [8,58,59,60,61]. Figure 5 shows stress responsive proteins, viz. superoxide dismutase (SOD), ascorbate peroxidase (APX), and catalase (CAT) were increased in carnation shoot cultures under hyperhydricity (−Si/+Hyperhydricity), whereas the expression levels were reduced when Si was supplied (+Si/+Hyperhydricity). The immunoblots expression of PsaA and PsbA significantly reduced their expressions (Figure 5) in hyperhydric carnation shoot cultures (−Si/+Hyperhydricity), but expression levels were the same in Si supplied shoot cultures (+Si/+Hyperhydricity) compared to control (−Si/−Hyperhydricity). Interestingly, the shoot cultures treated with Si alone (+Si/−Hyperhydricity), the PsaA and PsbA were expressed in higher amounts even than control (−Si/−Hyperhydricity). The results confirmed that Si supply to carnation shoot cultures can mitigate hyperhydricity to a greater extent.

This pioneering proteomic study was conducted to gain insights into the molecular basis of hyperhydric carnation shoot cultures to Si supply. About 900 proteins were identified and 70 of them differentially accumulated in response to hyperhydricity. The hyperhydricity (−Si/+Hyperhydricity) transiently suppresses the synthesis of proteins involved particularly in stress response, photosynthesis, and signal transduction; however, the supply of Si overpowers the negative effect of hyperhydricity in carnation shoot cultures.

### 2.5. Protein–Protein Interaction Network

**Figure 6:**
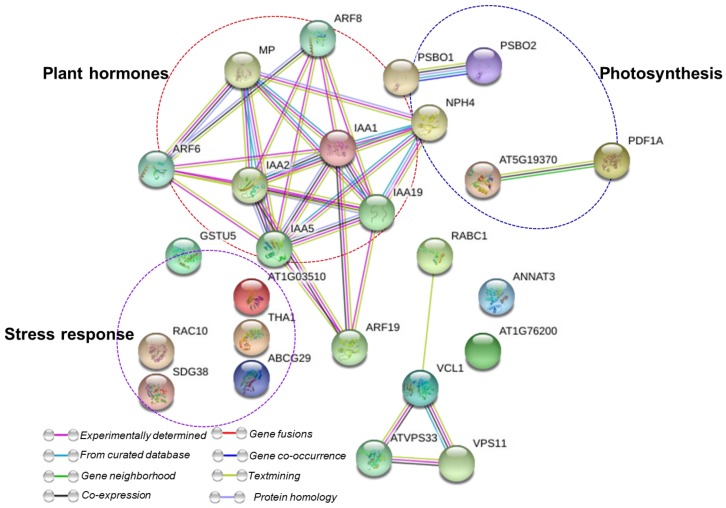
Analysis of protein interaction network by STRING 9.1. TAIR (the Arabidopsis information resource) homologous proteins from identified proteins were mapped by searching the STRING 9.1 software with a confidence of 0.4 using *Arabidopsis thaliana*. Colored lines between the proteins indicate the various types of interaction evidence. The clusters of highly interacting protein nodes are marked with oval dotted lines and include proteins involved in stress response, photosynthesis, and plant hormones.

The protein–protein interaction network generated using STRING 9.0 [62] discovered functional relations amid different proteins identified in carnation to hyperhydricity and silicon treatment (Figure 6). The key clusters of protein–protein interaction are highlighted in dotted circles. The interactions of proteins that were associated with stress response, photosynthesis, and plant hormones (part of the signaling process) were observed. The STRING [62] analysis of carnation exceptionally showed a higher interaction of plant hormone proteins, particularly auxin, than other functional categories of identified proteins. The protein–protein interaction results depict a greater chance of auxin production in carnation cultures under exogenous silicon treatment, which could facilitate cell division and differentiation even if they are under hyperhydricity, as also shown in our physiological parameters.

### 2.6. Ultrastructure of Chloroplasts

**Figure 7:**
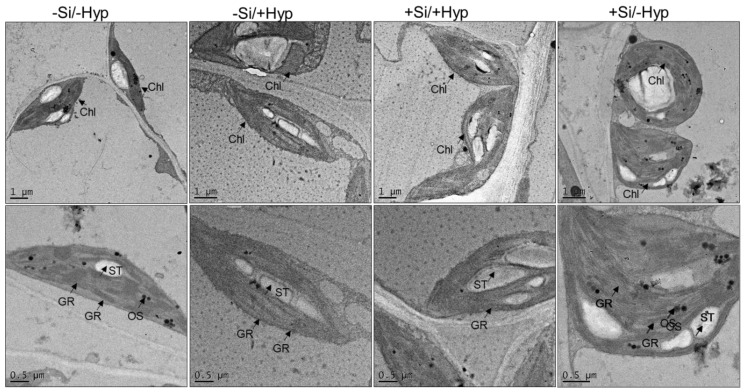
Ultrastructural changes in chloroplasts under hyperhydricity and silicon (Si) treatments (−Si/−Hyperhydricity; −Si/+Hyperhydricity; +Si/+Hyperhydricity; +Si/−Hyperhydricity) in shoot cultures of carnation (*Dianthus* *caryophyllus* L.) grown in vitro. In the figures, Chl denotes chloroplasts, GR denotes grana, ST indicates starch granules, and OS denotes osmium tetroxide.

Proteomic analysis showed a greater impact on photosynthetic proteins in carnation under hyperhydricity. Indeed, our morphological pictures showed some bigger changes in leaves such as brittleness, bushy appearances, and yellowness. Thus, it was necessary to observe the internal structure of leaf particularly chloroplasts (main organelle for photosynthesis). We accordingly observed interesting changes in chloroplast ultrastructure following transmission electron microscopy. The electron micrographs of normal carnation leaves showed organized thylakoid lamella and stroma (Figure 7), while disorganization of thylakoid lamella and grana was observed in hyperhydric leaves compared to control. Moreover, the decreased number of chloroplasts was also observed in hyperhydric (−Si/+Hyperhydricity) carnation leaves compared to control. In addition, the cell wall also seemed to be redundant in hyperhydric chloroplasts; however, it was recovered in leaves supplemented with silicon.

The changes in chloroplast ultrastructure under hyperhydricity in carnation were in agreement with our proteomic data particularly related to photosynthetic proteins. It is well documented that changes in chloroplast ultrastructure result in degradation of photosynthetic proteins exposed to biotic/or abiotic stress such as mineral deficiency [63] and metal toxicity [63]. These results were previously described in several plants under other abiotic stresses such as mineral deficiency [63] and metal toxicity [64]. On the other hand, changes in chloroplasts ultrastructure might also lead to a poor opening of stomata during transpiration as previously observed in electron micrographs of hyperhydric carnation [65]. The present electron micrographs of chloroplasts revealed that hyperhydricity resulted in the reduction of chloroplasts numbers and the disintegration of grana while silicon supply recovered those effects.

## 3. Materials and Methods

### 3.1. Plant Material, Culture Conditions and Treatments

The healthy shoots of carnation (*Dianthus caryophyllus*) var. “Purple Beauty” grown in greenhouse were used as the explant source. The explants were cultured on Murashige and Skoog (MS) medium [66] as described in our previous report [9].

To induce hyperhydricity, we followed our previous methods [9] and within four weeks the cultures with and without hyperhydricity were further sub-divided as follows: culture without silicon supply and hyperhydricity (−Si/−Hyperhydricity); cultures without Si supply but hyperhydric (−Si/+Hyperhydricity); cultures with silicon supply and hyperhydric (+Si/+Hyperhydricity); and cultures with Si supply with no hyperhydricity (+Si/−Hyperhydricity). Silicon (Si) in the form of potassium silicate (K_2_SiO_3_) was supplied directly in MS medium at the concentration of 100 μM. The elements were also balanced to remove excess potassium as described by Soundararajan et al. [65]. Subsequently, four week-old shoot cultures under four treatments (−Si/−Hyperhydricity; −Si/+Hyperhydricity; +Si/+Hyperhydricity; +Si/−Hyperhydricity) were analyzed for experimental analysis.

### 3.2. Si-Concentration Determination

Silicon was estimated in shoot cultures of carnation by inductively coupled plasma optical emission spectrometry, (ICP-OES, Thermo Elemental—IRIS Advantage, Thermo Fisher Scientific, Dreieich-Buchschlag, Germany) as described in our previous studies [26].

### 3.3. DAB and NBT Staining

To visualize H_2_O_2_ and O_2_^1–^ localization, leaves from all the treatments were immersed in a 1% solution of 3,3′-diaminobenzidine (DAB) (Sigma Aldrich, St. Louis, MO, USA) and a 0.1% solution of nitro-blue tetrazolium (NBT) (Sigma Aldrich), respectively, and were examined as previously described in our studies [23]. 

### 3.4. Protein Sample Preparation for 2-DE

About 100 mg of samples were homogenized in liquid nitrogen in precooled pestle and mortar. The proteins were extracted in a commercially available protein extraction buffer kit (Bio-Rad, Hercules, CA, USA) according to manufacturer’s instructions described in detail in our previous reports [9]. 

### 3.5. Two-Dimensional Gel Electrophoresis (2-DE) and Staining

For isoelectric focusing (IEF), the Multiphor^TM^ II system (GE Healthcare, Little Chalfont, UK) and IPG strip (pH 4–7, nonlinear, 13 cm, GE Healthcare) were used according to the manufacturer’s instructions, and the description is given in our previous studies [9]. The gels were stained in silver stain and procedure silver staining is described in detail in previous reports [9].

### 3.6. Image and Data Analysis

In each treatment, three independent biological replicates were taken. Gels were taken under constant settings by a photo imager and were examined as previously described [9].

### 3.7. Protein in Gel Digestion

The differential protein spots were excised manually from the 2D gels with the help of a clean razor blade and were chopped into small pieces. The description for in-gel digestion is given in detail in our previous reports [9].

### 3.8. Protein Identification Using MALDI-TOF MS and MS/MS Analysis

The digested peptide solution was spotted onto the MALDI-TOF MS target plate with a pipette. MALDI-MS analysis was performed with a Voyager DE-STR mass spectrometer (Applied Biosystems, Framingham, MA, USA). For a full description, please refer to our previous reports [9].

### 3.9. Protein Functional Classifications and Hierarchical Clustering

The identified proteins were classified into different categories of biological processes in which they are involved according to gene ontology [23] . Hierarchical clustering of differentially expressed protein spots was carried out using hierarchical clustering explorer (HCE 3.5 Interactive Power Analysis) software (Human-Computer Interaction Lab, University of Maryland, Baltimore, MD, USA).

### 3.10. Western Blots (Immunoblots)

For Western blotting, leaves were homogenized in extraction buffer as described previously [9]. The protein concentration was determined by the Bradford method using BSA (bovine serum albumin) as a standard curve. After electrophoresis, the gels were transferred to 0.45 μM nitrocellulose membrane (Sigma-Aldrich) and were further processed for Western blots as described in our previous studies [9]. The following primary antibodies were used: polyclonal primary antibodies 1:1000 dilution of anti PsaA (Agrisera # AS06 172) for PsaA, anti PsbA (Agrisera # AS05 084) for PsbA, anti-SOD (Cell Signaling #2770) for superoxide dismutase, 1:1000 dilution of anti-CAT (Cell Signaling #12980) for catalase, and 1:1000 dilution of anti-APX/L (Cell signaling #AS08 368) for ascorbate peroxidase. The blots were treated with 1:1000 dilution horseradish-linked anti-rabbit 1gG (Cell Signaling #7074) for 1 h as a secondary antibody. 

### 3.11. Protein–Protein Interactions

To determine the functions and interactions of the identified proteins, a protein–protein interaction network (PPI) was analyzed using the online tool STRING 9.0 (http://string-db.org).

### 3.12. Ultrastructure of Chloroplasts

For ultrastructures of chloroplasts, fresh leaves from shoot cultures were cut into small pieces approximately 2–3 mm^2^. Afterwards, the leaves were fixed in a fixative solution for 2–3 h in glutaraldehyde solution with pH 7.4. After fixation, the samples were dehydrated with ethanol series followed by embedding in epoxy resin. The further process for ultrastructure of chloroplast was done according to previous methods [22].

### 3.13. Statistical Analysis

Statistical analysis was performed as previously described in our studies [9].

## 4. Conclusions

The present study demonstrated numerous factors related to alleviation of hyperhydricity supplied with Si in carnation “Purple Beauty” grown in vitro. The morphological and physiological endeavors showed a tremendous change in hyperhydric carnation supplied with Si and aided Si a pro factor to recover or relieve hyperhydricity. The active involvement of Si in regulation of protein synthesis, photosynthesis and stress response showed a rapid recovery of carnation towards hyperhydricity. From these study, we concluded that Si abetted the micropropagated carnation to trigger and survive under hyperhydric conditions. Therefore, Si can be used as an essential element during in vitro propagation of carnation to obtain healthy and hyperhydric free plants.

## Figures and Tables

**Table 1:** Protein identification in carnation under Si and hyperhydric treatments analyzed by MALDI-TOF MS.

Spot No.	Protein Name	Plant Species	Accession Number	Mr Value	Calcu. *pI*/Exp. *pI*	Sequence Coverage
		**Stress Response**				
11	Small heat shock protein	*Triticum aestivum*	HS21C_WHEAT	26579	9.6/6.1	28
12	Putative pentatricopeptide repeat containing protein	*Arabidopsis thaliana*	PPR7_ARATH	47546	6.8/6.2	17
17	Glutathione S-transferase	*Arabidopsis thaliana*	GSTU5_ARATH	25984	5.4/5.9	31
33	Superoxide dismutase (CuZn)	*Picea abies*	SODC2_PICAB	2230	8.3/6.5	100
42	Probable disease resistance protein	*Arabidopsis thaliana*	DRL24_ARATH	81866	7.7/6.5	21
43	Catalase	*Solanum melongena*	CATA_SOLME	56584	6.8/6.6	26
58	Annexin D3	*Arabidopsis thaliana*	ANXD3_ARATH	36233	6.0/5.8	21
		**Photosynthesis**				
16	Ribulose bisphosphate carboxylase small chain, chloroplastic	*Chlamydomonas moewusii*	RBS_CHLMO	18896	9.4/5.3	29
18	ATP synthase epsilon chain, chloroplastic	*Pinus koraiensis*	ATPE_PINKO	15118	8.8/6.1	36
21	Oxygen evolving enhancer protein 1-2, chloroplastic	*Arabidopsis thaliana*	PSBO2_ARATH	34998	5.9/6.1	21
22	Oxygen evolving enhancer protein 1-2, chloroplastic	*Arabidopsis thaliana*	PSBO2_ARATH	34998	5.9/5.3	21
24	Ribulose bisphosphate carboxylase small chain, chloroplastic	*Chlamydomonas moewusii*	RBS_CHLMO	18896	9.4/6.0	42
37	Rhodanese like/PpiC domain containing protein 12, chloroplastic	*Arabidopsis thaliana*	STR12_ARATH	32959	8.6/6.6	31
40	Oxygen dependent coproporphyrinogen III oxidase, chloroplastic	*Hordeum vulgare*	HEM6_HORVU	43529	8.0/6.5	48
41	Photosystem I assembly protein	*Chaetosphaeridium globosum*	YCF4_CHAGL	21209	9.6/6.4	28
57	Oxygen evolving enhancer protein 1-2, chloroplastic	*Arabidopsis thaliana*	PSBO2_ARATH	34998	5.9/4.3	21
61	Oxygen evolving enhancer protein 1-1, chloroplastic	*Arabidopsis thaliana*	PSBO1_ARATH	35121	5.5/5.5	37
67	Ribulose bisphosphate carboxylase small chain, chloroplastic	*Chlamydomonas moewusii*	RBS_CHLMO	18896	9.4/5.7	36
70	Glucose-1-phosphate adenyl transferase large subunit 3, chloroplastic/amyloplastic	*Solanum tuberosum*	GLGL3_SOLTU	53569	8.9/5.8	24
		**Signal transduction**				
1	Peptide deformylase 1A, chloroplastic/mitochondrial	*Arabidopsis thaliana*	DEF1A_ARATH	29977	8.6/5.0	37
2	Ras related protein RABC1	*Arabidopsis thaliana*	RABC1_ARATH	23516	5.6/5.2	30
3	Guanine nucleotide-binding protein α-2 subunit	*Pisum sativum*	GPA2_PEA	44641	5.81/5.0	25
4	Ras related protein RABC1	*Arabidopsis thaliana*	RABC1_ARATH	23516	5.6/4.8	30
6	Ras related protein RABC1	*Arabidopsis thaliana*	RABC1_ARATH	23516	5.6/4.9	36
7	Glutathione *S*-transferase	*Arabidopsis thaliana*	GSTU5_ARATH	25984	5.4/4.8	36
10	Ras related protein RABC1	*Arabidopsis thaliana*	RABC1_ARATH	23516	5.6/6.0	30
13	Ras related protein RABC1	*Arabidopsis thaliana*	RABC1_ARATH	23516	5.6/6.3	30
27	Ras related protein RABC1	*Arabidopsis thaliana*	RABC1_ARATH	23516	5.6/6.3	31
30	Ras related protein RABC1	*Arabidopsis thaliana*	RABC1_ARATH	23516	5.6/6.3	31
31	Ras related protein RABC2	*Arabidopsis thaliana*	RABC1_ARATH	23517	5.6/6.4	32
		**Cell cycle/Cell division**				
5	Cell division cycle protein 48 homolog	*Capsicum annuum*	CDC48_CAPAN	89275	5.0/4.9	15
23	Cell division cycle protein 48 homolog	*Capsicum annuum*	CDC48_CAPAN	89275	5.0/5.9	17
26	Cell division cycle protein 48 homolog	*Capsicum annuum*	CDC48_CAPAN	89275	5.0/6.2	15
53	Cell division cycle protein 48 homolog	*Capsicum annuum*	CDC48_CAPAN	89275	5.0/6.8	18
54	Cell division cycle protein 48 homolog	*Capsicum annuum*	CDC48_CAPAN	89275	5.0/6.9	18
		**Transport**				
19	ABC transporter G family member 29	*Arabidopsis thaliana*	AB29G_ARATH	160195	8.5/6.2	9
25	ABC transporter G family member 29	*Arabidopsis thaliana*	AB29G_ARATH	160195	8.5/5.3	13
36	NADH dehydrogenase [ubiquinone] 1 β subcomplex subunit 2	*Arabidopsis thaliana*	NDUB2_ARATH	7563	8.9/6.3	69
47	NADH dehydrogenase [ubiquinone] 1 β subcomplex subunit	*Arabidopsis thaliana*	NDUB2_ARATH	7563	8.9/7.0	52
		**Plant hormone**				
32	Auxin responsive protein IAA1	*Arabidopsis thaliana*	IAA1_ARATH	19019	7.6/6.5	40
51	Auxin responsive protein IAA1	*Arabidopsis thaliana*	IAA1_ARATH	19019	7.6/7.0	40
		**Protein biosynthesis**				
8	tRNA(Ile)-lysidine synthase, chloroplastic	*Zygnema circumcarinatum*	TILS_ZYGCR	48914	9.8/4.9	20
14	30S ribosomal protein S11, chloroplastic	*Gnetum parvifolium*	RR11_GNEPA	14467	11.0/6.4	35
65	50S ribosomal protein L14, chloroplastic	*Brachypodium distachyon*	RK14_BRADI	13586	8.5/5.6	50
66	30S ribosomal protein S19, chloroplastic	*Platanus occidentalis*	RR19_PLAOC	10593	10.7/6.4	55
69	30S ribosomal protein S8, chloroplastic	*Acutodesmus obliquus*	RR8_ACUOB	15189	9.9/6.7	38
		**Secondary metabolism**				
9	Glycerol-3-phosphate dehydrogenase	*Oryza sativa*	GPDA_ORYSJ	46733	9.7/5.9	18
28	Tropinone reductase 1	*Datura stramonium*	TRN1_DATST	29598	6.1/6.4	18
46	Histone lysine N methyltransferase	*Arabidopsis thaliana*	ATXR4_ARATH	36111	6.3/6.8	35
52	Rac-like GTPbinding protein	*Arabidopsis thaliana*	RAC10_ARATH	23863	7.5/6.5	53
60	Probable low specificity l-threonine aldolase	*Arabidopsis thaliana*	THA1_ARATH	38917	6.9/5.6	42
		**Endosomal Transport**				
56	Protein VACUOLELESS1	*Arabidopsis thaliana*	VCL1_ARATH	96570	5.5/4.2	24
		**Pectin catabolic process**				
59	Pollen allergen Amb a 3	*Ambrosia artemisiifolia*	MPAA3_AMBEL	11368	6.1/5.6	59

## Supplementary Materials

Supplementary materials can be found at www.mdpi.com/1422-0067/19/1/50/s1.
