# Supplementary material for: Proteomic Analysis Reveals the Dynamic Role of Silicon in Alleviation of Hyperhydricity in Carnation Grown In Vitro
Source: Int J Mol Sci. 2017 Dec 24;19(1):50. doi: 10.3390/ijms19010050 (PMC5796000; doi:10.3390/ijms19010050)
Supplement: Supplementary file 1 [file ijms-19-00050-s001.pdf]

Supplementary Table 1. Spot quantification analyzed by Progenesis software under hyperhydricity and silicon (Si) treatments (-Si/-Hyperhydricity; -Si/+Hyperhydricity; +Si/+Hyperhydricity; +Si/-Hyperhydricity) in shoot cultures of carnation (*Dianthus caryophyllus* L.) grown *in vitro.*

**Condition**		**Condition 1**	**Condition 2**	**Condition 3**	**Condition 4**
**Replicates**		**3**	**3**	**3**	**3**
**Spot No.**	**Fold change**	**Average Normalized Volumes**			
		**-Si/-Hyp**	**-Si/+Hyp**	**+Si/+Hyp**	**+Si/-Hyp**
1	1.6	8041.002	5504.067	8930.403	8166.302
2	2.1	2603.602	1248.242	1776.58	2034.809
3	3.8	2408.332	4957.961	6288.096	1644.825
4	5.7	9252.312	1.19E+04	2.13E+04	3709.159
5	2.9	658.838	989.192	1894.148	862.395
6	10.5	933.487	2865.556	4849.969	461.338
7	4.6	266.71	725.141	157.945	268.191
8	2	8268.023	6734.306	4176.626	5376.128
9	4.8	207.971	351.068	653.154	1005.102
10	3.8	1.26E+05	4.76E+05	3.70E+05	1.77E+05
11	2.7	1.60E+04	1.10E+04	2.23E+04	8214.281
12	4.1	193.683	798.155	444.145	221.442
13	2.8	282.586	384.074	238.698	139.017
14	2.6	228.609	585.113	602.09	535.152
15	4.7	1666.94	2571.499	7878.23	3287.189
16	1.9	3121.147	4634.899	4783.466	2453.089
17	4.4	5088.136	7343.424	1.12E+04	2523.213
18	2.9	1898.724	2926.567	4131.499	1407.389
19	2.4	1.13E+04	9098.764	2.20E+04	1.61E+04
20	4.7	6356.599	1.93E+04	2.72E+04	5789.487
21	3.9	5.61E+04	8.45E+04	1.59E+05	2.19E+05
22	3.6	5264.356	6780.314	7361.644	1.87E+04

23	6.9	257.185	249.048	226.823	1556.248
24	2	1.54E+05	2.07E+05	3.12E+05	2.00E+05
25	5.5	1.48E+04	2.65E+04	3.57E+04	8.09E+04
26	3.3	1.38E+04	1.94E+04	3.07E+04	4.50E+04
27	2.1	231.784	279.054	191.196	394.906
28	2.6	8.21E+04	6.88E+04	6.48E+04	1.69E+05
29	2.8	2.38E+04	4.11E+04	6.56E+04	2.35E+04
30	4.2	5454.863	1.16E+04	1.88E+04	2.30E+04
31	2.8	7442.491	6617.283	1.55E+04	1.82E+04
32	8.4	1473.258	2598.504	1078.299	9038.538
33	1.6	2.00E+04	2.15E+04	2.07E+04	3.23E+04
34	1.2	1.11E+04	1.11E+04	1.25E+04	1.08E+04
35	1.7	6104.176	6783.315	6579.047	3918.299
36	2.8	9560.299	1.18E+04	1.78E+04	6366.467
37	1.9	1008.102	1350.262	1313.434	1892.102
38	5.8	82.553	69.013	71.253	402.287
39	7.4	5.65E+04	4.15E+04	7.62E+04	3.07E+05
40	4.4	1430.393	2480.481	3098.327	6244.674
41	4.1	1.49E+04	2.37E+04	5771.51	5868.222
42	3	1.73E+04	3.66E+04	5.21E+04	4.06E+04
43	3.2	2076.531	1450.281	640.091	1165.033
44	2.1	4.80E+04	3.81E+04	7.60E+04	3.62E+04
45	3.2	1724.092	1207.234	3804.921	2118.465
46	7.3	1.35E+04	1864.361	2658.932	7805.843
47	2.2	1.21E+04	7115.379	7526.714	1.53E+04
48	1.7	4.32E+04	4.12E+04	4.20E+04	2.50E+04
49	1.6	5.90E+04	4.39E+04	7.07E+04	5.15E+04
50	2.1	558.822	318.062	313.514	263.27
51	3	1.51E+04	1.07E+04	1.22E+04	4995.985

52	4	6137.515	7474.449	4787.028	1868.727
53	3.7	8.35E+04	1.14E+05	5.43E+04	3.05E+04
54	4.8	755.68	875.17	283.825	180.845
55	4.3	2.04E+04	2.25E+04	1.00E+04	5198.974
56	1.6	2.87E+04	2.87E+04	1.84E+04	2.20E+04
57	5.4	3.29E+04	7550.464	9804.442	4.05E+04
58	1.6	3137.023	2285.443	3193.331	1949.923
59	1.3	1.34E+04	1.50E+04	1.57E+04	1.79E+04
60	2.1	5.35E+04	4.75E+04	9.81E+04	9.41E+04
61	4.7	434.992	1058.205	558.15	223.903
62	3.6	4.57E+04	8.07E+04	1.66E+05	7.19E+04
63	4.6	3.64E+04	1.66E+05	9.56E+04	5.14E+04
64	2.6	5.65E+04	1.32E+05	1.48E+05	5.87E+04
65	7.4	4.85E+04	1.12E+05	1.72E+05	2.32E+04
66	3.3	987.464	3255.631	2446.36	1050.621
67	6.4	7712.377	2.56E+04	4.92E+04	8890.91
68	3.5	2359.117	675.131	787.348	1483.664
69	4.7	8857.009	1.65E+04	4.19E+04	1.12E+04
70	3.6	2.87E+04	7.83E+04	8.24E+04	2.28E+04
